# Serum metabolite markers of early *Mycoplasma hyopneumoniae* infection in pigs

**DOI:** 10.1186/s13567-019-0715-2

**Published:** 2019-11-26

**Authors:** Meera Surendran Nair, Dan Yao, Chi Chen, Maria Pieters

**Affiliations:** 1 Department of Veterinary Population Medicine, University of Minnesota, St. Paul, MN 55108 USA; 2 Department of Food Science and Nutrition, University of Minnesota, St. Paul, MN 55018 USA

## Abstract

*Mycoplasma hyopneumoniae*, the primary pathogenic bacterium causing enzootic pneumonia, significantly affects worldwide swine production. The infection is usually persistent and bacterial identification and isolation of *M. hyopneumoniae* in clinical samples are challenging due to the fastidious requirements for its growth. Hence, new practical surveillance tools that improve or complement existing diagnostics on *M. hyopneumoniae* are desirable, especially in early infection. The objective of this study was to identify potential metabolite markers of early *M. hyopneumoniae* infection in pigs through metabolomics analysis. Samples obtained from pigs in a previous *M. hyopneumoniae* experimental infection were used in this study. Briefly, two pigs served as mock inoculated controls and ten pigs were intra-tracheally inoculated with *M. hyopneumoniae*. Sera, laryngeal swabs (LS), and tracheo-bronchial lavage fluid (TBLF) were collected from all pigs at 0, 2, 5, 9, 14, 21 and 28 days post-inoculation (dpi). Bronchial swabs (BS) were collected post-mortem at 28 dpi. *Mycoplasma hyopneumoniae* infection was confirmed by PCR in LS, TBLF and BS. Serum metabolites were profiled using high-resolution liquid chromatography–mass spectrometry (LC–MS) analysis. Metabolite markers were identified by structural analysis following multivariate analysis of LC–MS data. The results showed that *M. hyopneumoniae* infection time-dependently altered the serum levels of selective amino acids and fatty acids. α-Aminobutyric acid and long-chain fatty acids were markedly increased at 14 and 21 dpi in inoculated pigs (*p* < 0.05). These results indicated that *M. hyopneumoniae* infection caused systemic changes in host metabolism, warranting further studies to determine underlying biochemical and physiological mechanisms responsible for the observed changes.

## Introduction

*Mycoplasma hyopneumoniae* (*M. hyopneumoniae*) is a major health risk in the porcine industry, causing enzootic pneumonia (EP), a chronic respiratory disease in growing pigs. Enzootic pneumoniae is often chronic, with a pattern of slow spreading and progression over the course of months, which usually results in herd morbidities that can reach up to 70–100% [1–3]. Coughing, poor weight gain, and reduced feed conversion are typical signs of infected pigs [1]. The incubation period of *M. hyopneumoniae* is difficult to predict under natural conditions as it may depend on the infectious dose, immune status of pigs, and presence of associated secondary respiratory infections [4]. However, in clinically affected herds, seroconversion and coughing have been reported to appear 2–6 weeks post-infection [5], varying across production systems. The prevalence of *M. hyopneumoniae* is often reported highest in growing-finishing pigs, although clinical disease or pathogen detection can also occur early in nursery pigs and in breed-to-wean farms [6, 7].

Chronicity is a prominent characteristic of mycoplasma infections. The ability of the bacteria to escape detection by adaptive immune surveillance mechanisms is associated with the challenges in early detection and prevention [8]. In swine production, extended shedding and prolonged transmissibility of *M. hyopneumoniae* to naïve contact pigs has been documented up to 214 days post-infection (dpi) [9]. Because of low mortality associated with EP, post-mortem lung lesions are usually observed in slaughtered pigs or when losses occur due to superimposed secondary infections [10].

The fastidious growth requirements pose challenges for bacterial culture and isolation of *M. hyopneumoniae* from clinical samples. At present, serological assays targeting antibodies against *M. hyopneumoniae* are most commonly used to detect exposure [11]. While advantageous in cost and convenience, these assays have limitations, including low sensitivity to detect early or subclinical infection, potential antigenic cross-reactivity with other respiratory commensal mycoplasmas, and lack of discrimination between infected and vaccinated pigs, which count for more than 70% of pig herds globally [2]. Compared to serodiagnosis, PCR assays offer higher degree of accuracy in detecting the genomic DNA of *M. hyopneumoniae* [12] from clinical samples [13, 14]. However, the consistency of PCR detection across different sample types varies [13] and many sampling methods are considered invasive in live animals. All these situations make the diagnosis of *M. hyopneumoniae* infections extremely challenging.

Metabolomics has been utilized to identify dormant and intricate interactions between hosts and pathogens [15, 16]. Metabolic events that occur during host–pathogen interactions reflect how the host responds to pathogens and also how a pathogen adapts and proliferates in its host environment [15]. Applications of metabolomics in studying infectious diseases in humans and animals have unraveled novel knowledge about biochemical and physiological processes in virus, bacteria, and parasite infections [17–19], which could guide the identification of diagnostic biomarkers. To the best of our knowledge, metabolomics tools have not been employed to study the host responses to *M. hyopneumoniae* infection. In order to identify the metabolic changes associated with an early *M. hyopneumoniae* infection, our current study characterized the metabolic differences between infected and uninfected pigs through metabolomics analysis.

## Materials and methods

### Chemicals and reagents

Amino acid standards, α-aminobutyric acid, n-butanol, and sodium pyruvate were purchased from Sigma-Aldrich (St. Louis, MO, USA), LC–MS-grade water, acetonitrile (ACN), and formic acid were obtained from Fisher Scientific (Houston, TX, USA), 2,2′-dipyridyl disulfide (DPDS) was obtained from MP Biomedicals (Santa Ana, CA, USA), dansyl chloride (DC) was purchased from Acros Organics (Morris Plains, NJ, USA), 2-hydrazinoquinoline (HQ) and triphenylphosphine (TPP) were obtained from Alfa Aesar (Haverhill, MA, USA), and *p*-chlorophenylalanine was purchased from Alexis Biochemicals (San Diego, CA, USA).

### Experimental design

Samples for metabolomics analysis were obtained from 12 randomly-selected pigs that were either mock inoculated controls or inoculated with *M. hyopneumoniae* in an experimental study previously conducted by our research group [13]. At 0 dpi, 2 mock inoculated controls were intra-tracheally inoculated with 10 mL of sterile modified Friis medium [20], whereas the remaining 10 pigs were intra-tracheally inoculated with 10 mL of a lung homogenate containing 1 × 10^5^ CCU/mL of *M. hyopneumoniae* strain 232 [21]. Serum samples, laryngeal swabs (LS), and tracheobronchial lavage fluid (TBLF) collected on 0, 2, 5, 9, 14, 21 and 28 dpi were used for the analysis in the present study. At 28 dpi all pigs were euthanized, bronchial swabs (BS) were collected, and the level of lung lesions observed in each lobe were recorded in percentage (0 to 100%) as previously described [22].

### Detection of *M. hyopneumoniae* genetic material

Laryngeal swabs, TBLF, and BS samples were examined by a *M. hyopneumoniae* species specific real-time PCR [12]. Genomic DNA was extracted from samples using the DNeasy Blood and Tissue Kit (Qiagen, Valencia, CA, USA). Real-time PCR was performed using *M. hyopneumoniae* specific reagents and controls (VetMAX™, Life Technologies Corporation, Carlsbad, CA, USA). Samples were considered positive for detection of *M. hyopneumoniae* when the Ct value was < 37.

### Metabolomics analysis

Serum samples were analyzed using a liquid chromatography–mass spectroscopy (LC–MS) based metabolomics platform, which included serum sample preparation, chemical derivatization, data deconvolution processing, and multivariate analysis (MDA) followed by marker characterization and quantification [23].

#### Sample preparation

For detecting metabolites containing amino functional groups in their structure, samples were derivatized with DC prior to the LC–MS analysis. Briefly, 5 μL of serum or standard was mixed with 5 μL of 50 μmol/L d_5_-tryptophan (internal standard), 50 μL of 10 mmol/L sodium carbonate, and 100 μL of DC solution (3 mg/mL in acetone). The mixture was incubated at 60 °C for 15 min and centrifuged at 21 000 × *g* for 10 min, and the supernatant was transferred into a sample vial for LC–MS analysis. Additionally, to detect carboxylic acids, aldehydes, and ketones, samples were derivatized separately with HQ prior to the LC–MS analysis [24]. For HQ reactions, 2 μL of sample was added into a 100 μL of freshly prepared ACN solution containing 1 mM DPDS, 1 mM TPP, and 1 mM HQ. The reaction mixture was incubated at 60 °C for 30 min and then chilled on ice immediately. After centrifugation at 21 000 × *g* for 10 min, the supernatant was transferred into a high-performance liquid chromatography (HPLC) vial for LC–MS analysis.

#### LC–MS analysis

A 5 μL aliquot of each sample was injected into an ultraperformance liquid chromatography–quadrupole time-of-flight mass spectrometry (UPLC–QTOFMS) system (Waters Corporation, Milford, MA, USA) and separated by a BEH C18 column (Waters Corporation) with a gradient of mobile phase ranging from water to 95% aqueous ACN containing 0.1% formic acid over a 10-min run. Capillary voltage and cone voltage for electrospray ionization were maintained at 3 kV and 30 V for positive mode detection, whereas source temperature and desolvation temperature were set at 120 and 350 °C, respectively. Nitrogen was used as both cone gas (50 L/h) and desolvation gas (600 L/h), and argon was used as collision gas. For accurate mass measurement, the mass spectrometer was calibrated with sodium formate solution (range *m/z* 50 to 1000) and monitored by the intermittent injection of the lock mass leucine enkephalin ([M+H]^+^ = 556.2771 m/z) in real time. Mass chromatograms and mass spectral data were acquired and processed by MassLynx software (Waters Corporation) in centroided format. Additional structural information was obtained by tandem MS (MS/MS) fragmentation with collision energies ranging from 15 to 40 eV [25].

#### Marker identification and characterization

The chromatographic and spectral data of samples from the UPLC–QTOFMS system were deconvoluted using MarkerLynx software (Waters Corporation). MarkerLynx provided a multivariate data matrix containing information on sample identity, ion identity (retention time and m/z), and ion abundance. The abundance of each ion was calculated by normalizing the single ion counts to the total ion counts in the entire chromatogram, and the data matrix was then exported into SIMCA-P+ software (Umetrics, Kinnelon, NJ, USA). Principal components analysis (PCA) was used to model the data for the mock inoculated controls and inoculated groups. Metabolite markers were identified by analyzing ions contributing to sample separation in PCA models. QuanLynx software (Waters Corporation) was used for the quantification of metabolites, and standard curves were generated by incorporating amino acid and fatty acid standard molecules in the LC–MS run. The concentrations or relative abundances of identified metabolite markers in the samples and correlations among these metabolite markers were evaluated using GraphPad and R program [26].

### Statistical analysis

Statistical analysis of metabolomic parameters was performed as a two-tailed Student’s *t* tests for paired data within a time point. Friedman test, the non-parametric alternative to the one-way ANOVA with repeated measures, was used to test for differences among metabolites between mock inoculated controls and inoculated pigs over time. Hierarchical cluster analysis (HCA) was performed to identify metabolite clusters contributing to sample separation. Pearson analysis was performed to evaluate the correlation between metabolite markers and detection of *M. hyopneumoniae* in serum samples. Differences between the mock inoculated controls and inoculated pigs were considered significant if *p* < 0.05 and were considered a trend when between 0.05 and 0.10.

## Results

### Detection of *M. hyopneumoniae* genetic material

**Figure 1:**
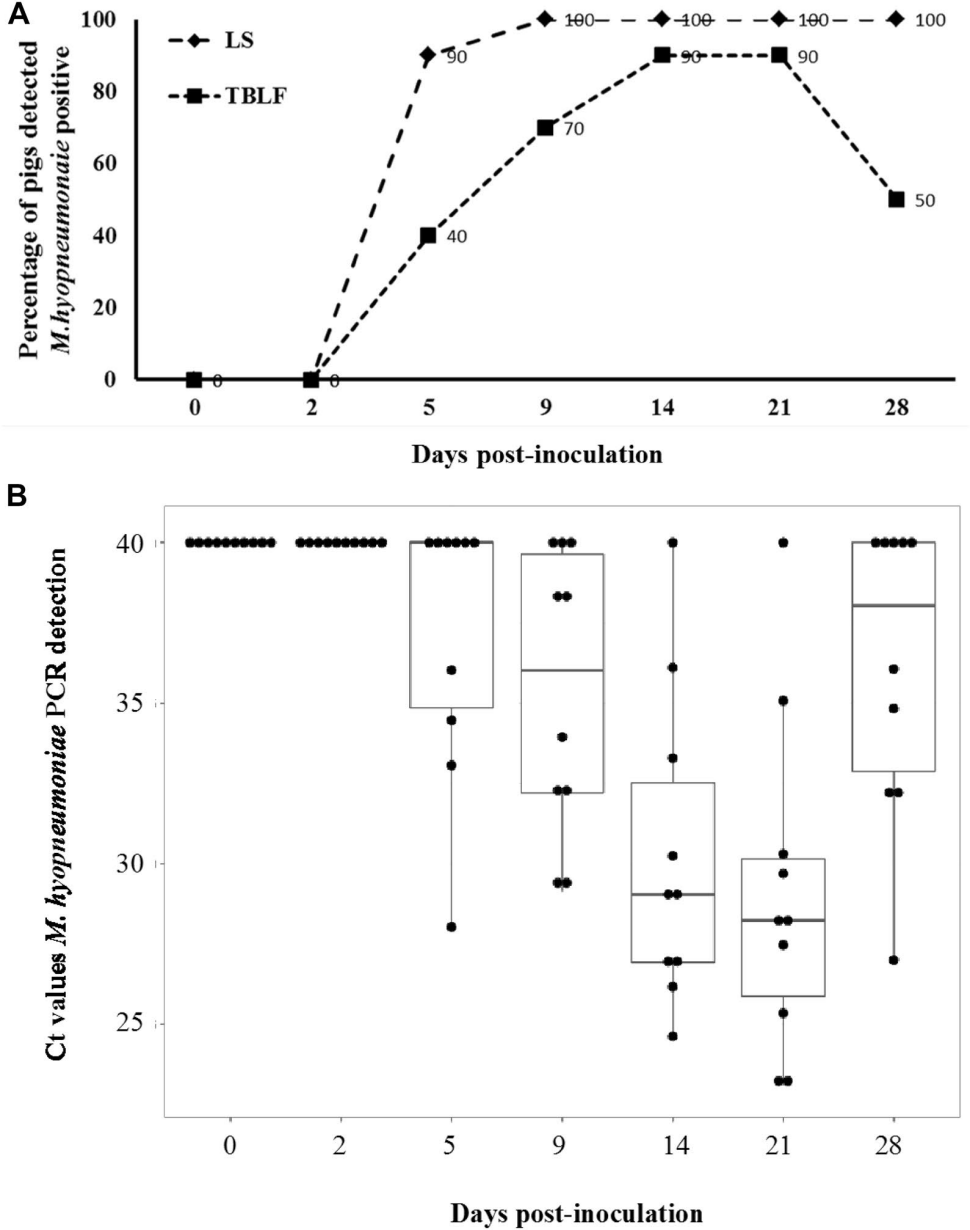
**Detection of** ***Mycoplasma hyopneumoniae*** **genetic material in laryngeal swabs and tracheobronchial lavage fluid. A** Percentage of pigs detected *M. hyopneumoniae*-positive in laryngeal swabs (LS) and tracheobronchial lavage fluid (TBLF) by real-time PCR. The LS and TBLF were obtained on seven samplings during the 28-day period following experimental inoculation with *M. hyopneumoniae*. The percentage of pigs detected positive for *M. hyopneumoniae* in TBLF was significantly higher at 14 and 21 dpi (*p* < 0.05). **B** Individual Ct values and mean from PCR detection of *M. hyopneumoniae* in TBLF samples from 10 inoculated pigs at different days post-inoculation. The Ct values of *M. hyopneumoniae*-negative samples were presented as 40, which is the arbitrary threshold value of *M. hyopneumoniae* PCR detection.

Real-time PCR analysis of LS, TBLF, and BS confirmed that mock inoculated control pigs were negative for *M. hyopneumoniae* throughout the study. Among inoculated pigs, *M. hyopneumoniae* DNA was detected in LS and TBLF of all pigs (100%) starting at 9 dpi (Figure 1A). The load of *M. hyopneumoniae* DNA in TBLF peaked at 14 and 21 dpi and then decreased at 28 dpi (Figure 1B). In addition, BS samples of all inoculated pigs at euthanasia (28 dpi) were positive for *M. hyopneumoniae* DNA.

### Lung lesions suggestive of *M. hyopneumoniae* infection

Post-mortem scoring of lung lesions was conducted at 28 dpi. Lesion scores ranged from 4 to 52% among the 10 inoculated pigs, with a mean score of 18.3%. No macroscopic lung lesions were observed in mock inoculated control pigs.

### Changes in serum metabolome during early *M. hyopneumoniae* infection

**Figure 2:**
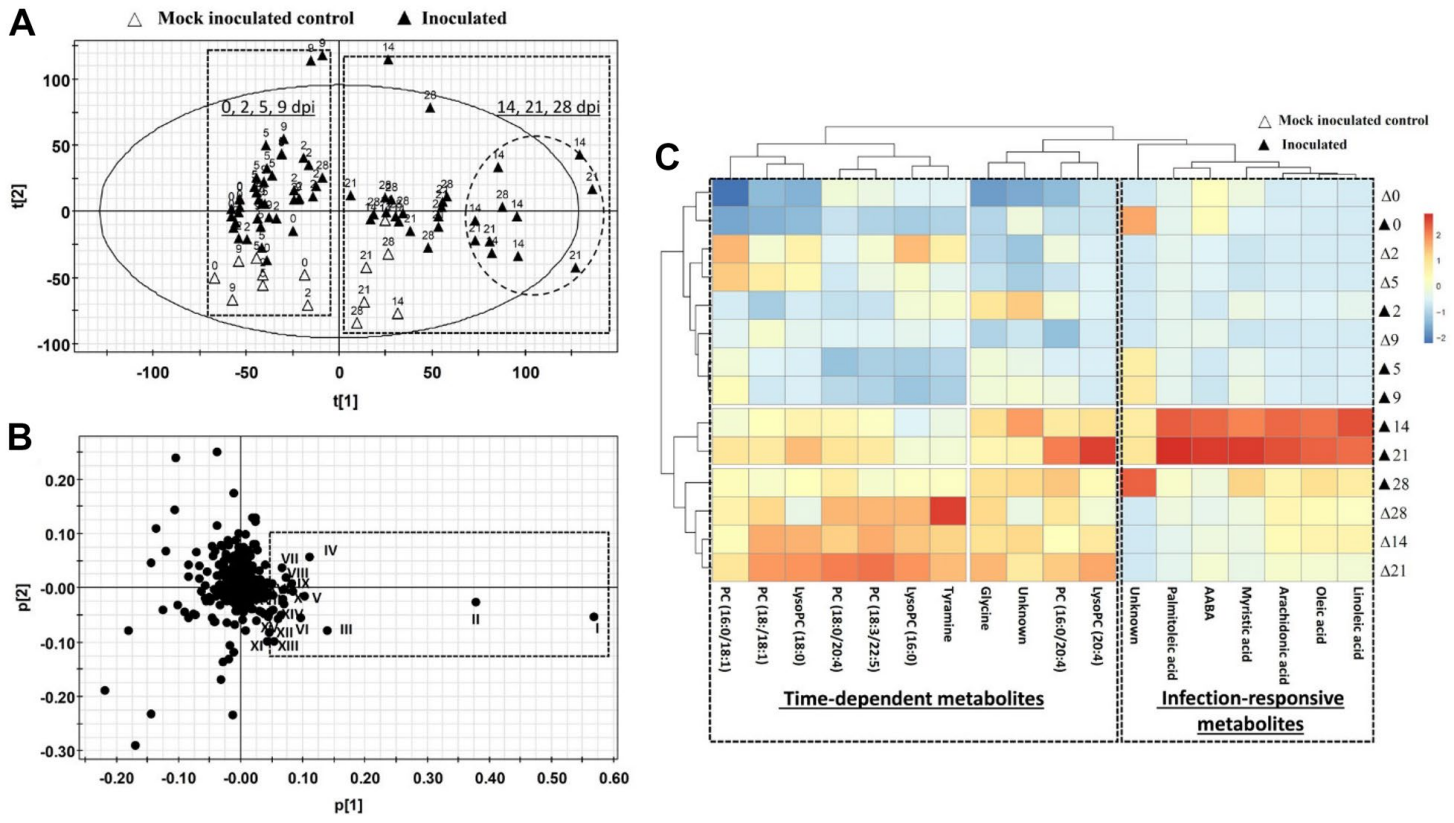
**Effects of** ***Mycoplasma hyopneumoniae*** **infection in serum metabolome.** Data from LC–MS analysis of serum samples were processed by principal component analysis (PCA). **A** Score plot of PCA model. Serum samples collected at 0, 2, 5, 9 dpi were separated from those collected at 14, 21, 28 dpi along the 1st principal component of the model (dashed boxes). Additionally, samples from inoculated pigs at 14 and 21 dpi were further separated from other samples (circled). **B** Loading plot of PCA model. Major markers contributing to sample separation (I–XX) in the PCA model were identified and labeled in the loading plot and further characterized by structural analysis. The identities of these metabolites are presented in Table 1. **C** Distribution profile of metabolite markers. The heat map from hierarchical cluster analysis classified the metabolite markers as time-dependent metabolites and infection-responsive metabolites.

The distribution of 84 serum samples from mock inoculated control and inoculated pigs in a score plot of the PCA model on pooled LC–MS data showed that samples collected at 0, 2, 5, and 9 dpi were clearly separated from samples collected at 14, 21, and 28 dpi along the 1st principal component of the model (Figure 2A). This distribution profile suggests the existence of time-dependent metabolic changes in both mock inoculated control and inoculated pigs. More importantly, the majority of serum samples from infected pigs at 14 and 21 dpi were further separated from other samples in the 1st principal component of the model, indicating the occurrence of infection-responsive metabolic changes (Figure 2A). The metabolites contributing to time- and infection-dependent sample separation were identified in a loading plot of the PCA model (Figure 2B), and their identities (I–XX) were elucidated as free fatty acids, phospholipids, and amino acid metabolites, respectively (Table 1).

**Table 1:** **Identities of top 20 metabolites associated with pig growth and** ***Mycoplasma hyopneumoniae*** **infection**

ID	Identity (derivative)	*m/z* of charged ion	Formula of original molecule	Database
I	Oleic acid (HQ)	424.3321+	C18H34O2	HMDB00207
II	Linoleic aicd (HQ)	422.3164+	C18H32O2	HMDB00673
III	PC(18:0/20:4)	810.6034+	C46H84NO8P	HMDB0013420
IV	Glycine (DC)	309.0906+	C2H5NO2	HMDB0000123
V	Palmitoleic acid	396.3009−	C16H30O2	HMDB0003229
VI	PC(18:0/18:1)	788.6193+	C44H86NO8P	HMDB08069
VII	PC(16:0/18:1)	760.5874+	C42H82NO8P	HMDB08315
VIII	PC(16:0/20:4)	782.5718+	C44H80NO8P	HMDB11221
IX	Myristic acid (HQ)	370.2851+	C14H28O2	HMDB00806
X	Arachidonic acid (HQ)	446.3167+	C20H32O2	HMDB01043
XI	LysoPC(16:0) (DC)	496.3405+	C26H42N6O5	
XII	Tyramine (DC)	371.1428+	C8H11NO	HMDB00306
XIII	Unknown (HQ)	279.0934+		
XIV	Alpha aminobutyric acid (DC)	337.1217+	C4H9NO2	HMDB01906
XV	LysoPC(18:0) (DC)	522.3562+	C14H36N6	HMDB39491
XVI	Unknown (DC)	365.1363+		
XVII	N_2_-Acetyl-l-ornithine (DC)	408.1588+	C7H14N2O3	HMDB0003357
XVIII	LysoPC(20:4) (HQ)	544.3401+	C20H38O2	
X1X	PC(O-20:0/21:0)	846.7581+	C50H99O12P	
XX	PC(18:3/22:5)	836.62+	C48H86NO8P	HMDB08056

The correlations between metabolites and samples were further characterized by hierarchical clustering analysis. Consistent with their distribution profile in the PCA model, samples at 0, 2, 5 and 9 dpi and samples at 14, 21, and 28 dpi formed two major clusters based on time (Figure 2C). The metabolites contributing to this time-dependent clustering are mainly comprised of various phosphatidylcholines (PC) and lyso-phosphatidylcholines (lyso-PC; Figure 2C). More importantly, multiple free fatty acids and an amino acid metabolites were identified as infection-responsive metabolites because these metabolites were present in much higher abundance in inoculated pig serum compared to mock inoculated control serum at 14 and 21 dpi (Figure 2C).

### Changes in serum free fatty acids during early *M. hyopneumoniae* infection

**Figure 3:**
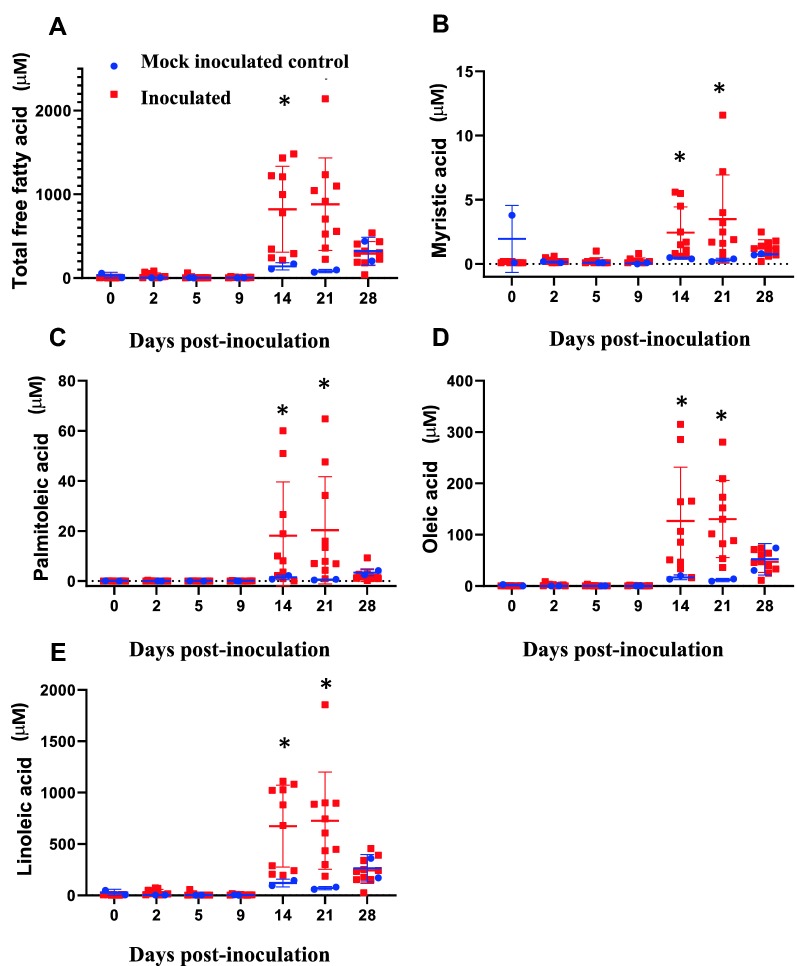
**Concentrations of free fatty acids in serum samples.** Concentrations of total and individual free fatty acids (FFA) in mock inoculated control and inoculated serum samples were measured. **A** Total FFA. **B** Myristic acid. **C** Palmitoleic acid. **D** Oleic acid. **E** Linoleic acid. *p < 0.05.

The concentrations of total free fatty acids (FFA) and individual FFA, including myristic acid, palmitoleic acid, oleic acid, and linoleic acid in serum samples were significantly elevated at 14 and 21 dpi in inoculated pigs (*p* < 0.05; Figures 3A–E).

### Changes in serum amino acids during early *M. hyopneumoniae* infection

**Figure 4:**
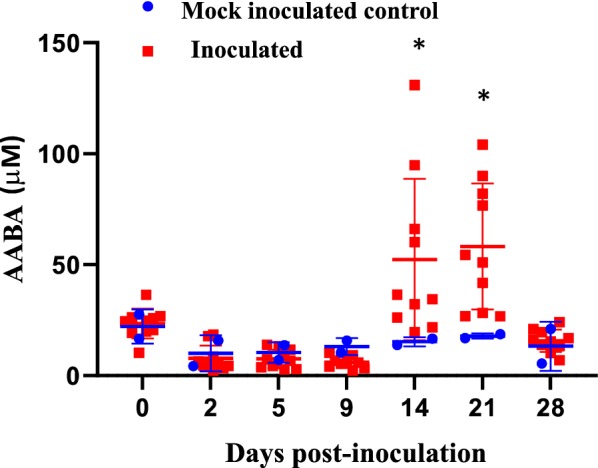
**Concentration of α-amino butyric acid (AABA) in serum samples.** Concentrations of α-amino butyric acid in mock inoculated control and inoculated serum samples were measured. *p < 0.05.

Quantitative analysis of free amino acids was conducted to determine the status of the serum amino acid pool. A significant increase in α-aminobutyric acid (AABA), a non-proteinogenic amino acid, was identified at 14 and 21 dpi in inoculated pigs (*p* < 0.05; Figure 4). The quantified total free amino acids (FAA) in serum were comparable at all samplings between mock inoculated controls and inoculated pigs (data not shown).

### Correlation between metabolite markers and detection of *M. hyopneumoniae* in TBLF

**Figure 5:**
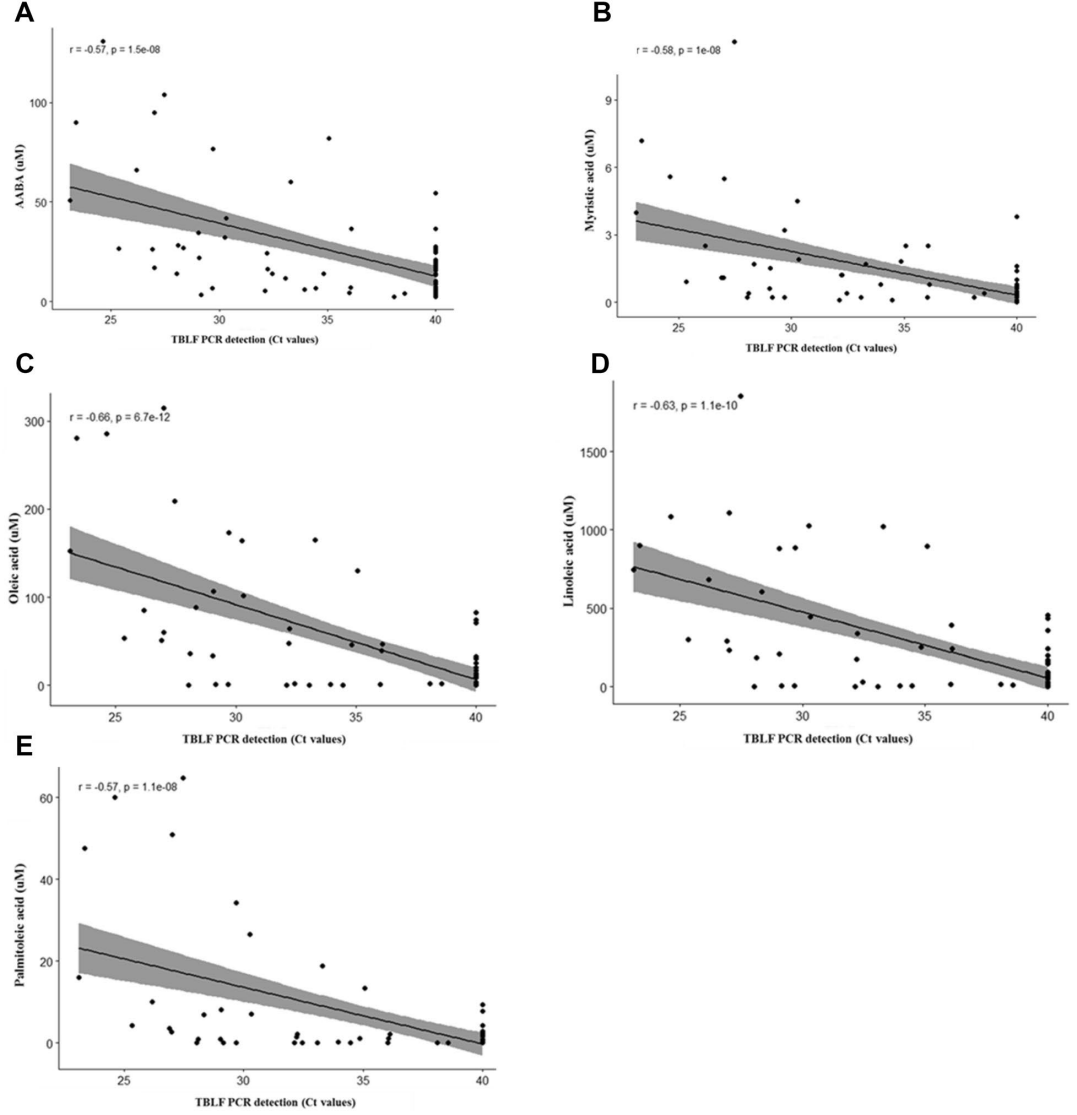
**Correlation between metabolite markers and detection of** ***Mycoplasma hyopneumoniae*** **in TBLF. A** Alpha-amino butyric acid concentrations were strongly correlated with *M. hyopneumoniae* PCR detection patterns in TBLF (r = −0.57; *p* < 0.05). **B** Long chain fatty acid concentrations were strongly correlated with *M. hyopneumoniae* PCR detection patterns in TBLF (r ≥ 0.5; *p* < 0.05).

A significant correlation was observed between the concentration of AABA detected in serum of inoculated pigs and Ct values for *M. hyopneumoniae* detection in TBLF (*p* < 0.05). A negative correlation was indicated based on low Ct values (high bacterial burden and greater correlation with the increased concentration of AABA; Figure 5A). A similar effect was also observed with the long chain fatty acid concentrations and Ct values (*p* < 0.05; Figures 5B–E).

## Discussion

*Mycoplasma hyopneumoniae* has been recognized as an endemic pathogen globally. The typical lesions of EP have been reported to occur in 30 to 80% of slaughter pigs in different countries [20]. In the United States alone, particularly, in large swine operations with more than 5000 pigs, 28.3% of nursery sites and 57.4% of finisher sites have been reported positive for *M. hyopneumoniae* in a survey conducted by the National Animal Health Monitoring System [27]. The lack of clear understanding on the pathogenesis of *M. hyopneumoniae* limits current efforts to control and manage the clinical disease in the field. Furthermore, the mechanisms of immune modulation and the immunopathology associated with EP are not fully understood [3]. Hence, there is a growing need for practical surveillance tools to complement diagnostics to demonstrate early or sub-clinical infections in swine populations. In this study, potential candidate biomarkers in serum associated with pathophysiological events occurring in early *M. hyopneumoniae* infections were identified using state-of-art metabolomics analysis.

The serum metabolomics profile identified both, infection-dependent and independent changes in experimentally infected pigs. The host metabolic responses to *M. hyopneumoniae* challenge were observed as early as 5 dpi. It has been shown that the attachment of bacterial cells to tracheal cilia is one of the events preceding *M. hyopneumoniae* infection and it may occur 5 days after experimental challenge [28], when most pigs remain asymptomatic [11, 29, 30].

A significant increase in the levels of AABA was identified at 14 and 21 dpi in this study. AABA is a non-proteogenic amino acid and often considered to be a catabolic product of other amino acids. As shown in humans, AABA is biosynthesized by transamination of α-ketobutyric acid, a metabolite in branched chain amino acids biosynthesis [31]. Alternatively, α-ketobutyric acid is decarboxylated to propionate with the formation of propionyl-CoA and succinyl-CoA, and enters the Krebs cycle [32, 33]. Ophthalmic acid is the down-stream byproduct from AABA and is known to be an oxidative stress marker involved in glutathione metabolism [34]. Observing the significant changes in AABA levels in inoculated pigs, the abundances of the precursor, α-ketobutyric acid and the downstream product, ophthalmic acid were also evaluated in this study. However, there were no marked changes observed in the concentrations of the two metabolites in the serum of inoculated pigs compared to the mock inoculated controls.

Several studies have also reported AABA as a metabolic indicator of disease severity in human patients with sepsis, as well as multiple organ failure syndrome [35, 36]. Increases in AABA have been suggested to be caused by protein hypercatabolism with enhanced intravascular release of endogenous amino acids to circulation and liver dysfunction. Conversely, the role of AABA in infectious disease metabolism in swine has not been determined in detail yet.

The levels of FFA in serum samples were elevated at 14 and 21 dpi in inoculated pigs. This observation is consistent with the reported lipolytic activity of mycoplasmas [37]. The p65 of *M. hyopneumoniae* is a surface lipoprotein which has recently been identified to exhibit lipolytic activity with affinity to a wide range of fatty acid esters. Schmidt et al. speculated that the lipolytic function of p65 could be the reason of the reduced function of surfactants in pneumonic pig lungs [37]. Pulmonary surfactants are surface-active complex mixtures consisting of phospholipids, neutral lipids, and specific proteins. Surfactants are essential for normal lung function by reducing surface tension at pulmonary air–liquid interfaces in animals and humans [38, 39]. Among surfactant phospholipids, dipalmitoyl phosphatidylcholine is reported to be the principal component that reduces minimal surface tension at end-expiration [40]. Genome scale modelling studies have revealed surfactant degradation and myo-inositol catabolism as critical traits for virulence in *M. hyopneumoniae* [41].

The nutritional requirement of *Mycoplasma* organisms for fatty acids is well known since their discovery. The fastidious nature of mycoplasmas in vitro is defined by the requirement of a exogenous supply of amino acids and fatty acids in their growth medium [39]. A majority of *Mycoplasma* species including *M. hyopneumoniae*, lack the genes involved in fatty acid synthesis and therefore, the building units for bacterial membrane lipids are obtained from long chain saturated and unsaturated fatty acids present in the host [8]. Fatty acid analyses have revealed that Cl6–Cl8 fatty acids account for 79% of the *M. hyopneumoniae* membrane lipid composition, of which oleic, as well as palmitic acids are the major fatty acids [41]. Recently, metabolic modelling studies have suggested that an exogenous supply of fatty acids such as palmitic, stearic, oleic and linoleic acids could improve the proliferation and survival of *M. hyopneumoniae* both in vitro and in vivo [39].

The present study identified significant changes in the host amino acid and fatty acid profiles during early stages of *M. hyopneumoniae* infection. The requirement of fatty acids by the pathogen was justified by the observed increased abundance of long chain fatty acids in the serum. The increased levels of AABA along with altered kinetics of amino acids may indicate protein hyper-catabolism in hosts. It is also important to note that the host metabolic signature in response to *M. hyopneumoniae* infection reflected the active metabolic pattern of the pathogen, rather than that of the defense mechanisms. Thus, through characterizing the metabolic signature of *M. hyopneumoniae* infection in pigs, the study examined the metabolic responses to early disease which was sparsely reported before. The information procured would aid to improve the capabilities to diagnose, control, and eradicate this bacterium from swine herds. Nevertheless, the changes observed in the experimental inoculation need to be further validated in the natural course of infection. Moreover, the molecular mechanisms involved in host responses to *M. hyopneumoniae* infection need to be deciphered.
